# A Comprehensive Analysis of MicroRNAs in Human Osteoporosis

**DOI:** 10.3389/fendo.2020.516213

**Published:** 2020-10-21

**Authors:** Ying Huai, Wenjuan Zhang, Zhihao Chen, Fan Zhao, Wei Wang, Kai Dang, Kaiyue Xue, Yongguang Gao, Shanfeng Jiang, Zhiping Miao, Meng Li, Qiang Hao, Chu Chen, Airong Qian

**Affiliations:** ^1^ Lab for Bone Metabolism, Key Lab for Space Biosciences and Biotechnology, School of Life Sciences, Northwestern Polytechnical University, Xi’an, China; ^2^ Research Center for Special Medicine and Health Systems Engineering, School of Life Sciences, Northwestern Polytechnical University, Xi’an, China; ^3^ NPU-UAB Joint Laboratory for Bone Metabolism, School of Life Sciences, Northwestern Polytechnical University, Xi’an, China; ^4^ State Key Laboratory of Cancer Biology, Biotechnology Center, School of Pharmacy, Fourth Military Medical University, Xi’an, China; ^5^ Clinical Laboratory of Honghui Hospital, Xi’an JiaoTong University College of Medicine, Xi’an, China

**Keywords:** osteoporosis, microRNAs, bioinformatics analysis, biomarker, target genes

## Abstract

MicroRNAs (miRNAs) are single-stranded RNA molecules that control gene expression in various processes, such as cancers, Alzheimer’s disease, and bone metabolic diseases. However, the regulatory roles of miRNAs in osteoporosis have not been systematically analyzed. Here, we performed a comprehensive analysis to identify the differentially expressed miRNAs involved in osteoporosis. MiRNAs associated with osteoporosis were collected through literature retrieval and further screened based on specific inclusion and exclusion criteria. The osteoporosis therapeutic targets of miRNAs were obtained by the integration of miRWalk 3.0 database and five human disease therapeutic target databases. Then, the network analysis and functional enrichment analysis of miRNAs and their targets were performed. As a result, 11 eligible miRNAs were identified highly associated with osteoporosis. MiRNA-mRNA network demonstrated there were the complex mutual interactions between miRNAs and their targets. Besides, ADRB2, AR, ESR1, FGFR1, TRAF6, etc., were identified as the top hub genes in protein-protein interaction (PPI) network. Functional enrichment analysis revealed that miRNAs and their targets were mainly mapped on processes associated with bone and immune system, such as bone remolding, bone mineralization, PI3K/AKt, TNF signaling pathways and Th17 cell differentiation. RT-PCR results showed that the expression of miR-335-3p was significantly down-regulated in hind limb unloading (HLU) mice tibia samples compared with controls, the remaining 10 miRNAs were significantly up-regulated after HLU (*P* < 0.01). In summary, we identified 11 differentially expressed miRNAs and their hub target genes in osteoporosis, which may be novel diagnostic biomarkers for osteoporosis.

## Background

Osteoporosis is a subclinical chronic bone metabolic disease characterized by low bone mineral density (BMD), progressive bone loss, and destroyed bone microstructure. The typical signs and symptoms of osteoporosis mainly include pain, stiffness, skeletal hypofunction, and limitation of movement, even companied by an increased risk of fractures (1). Recently, osteoporosis has been aroused considerable attention all over the world given the growing morbidity and disability as well as the substantial cost to health care and social services worldwide (2). According to World Health Organization (WHO), more than 69.4 million people > 50 years are hampered by osteoporosis and approximately 687,000 populations develop to osteoporotic fractures each year each year in China (3). In US, it has been estimated that the financial costs associated with bone fractures will reach $25.3 billion by the end of 2025 (4). Therefore, accurate early diagnosis of onset is the key to the prevention and effective therapy for osteoporosis. If increased risk of bone loss is diagnosed prior to the first occurrence of osteoporosis, the morbidity may be significantly reduced by preventive pharmacologic treatments and/or lifestyle interventions. Presently, despite that BMD measurement by dual-energy X-ray absorptiometry (DXA) is the most recommended tool for osteoporosis risk monitoring, it involves complex equipment, which is bulky, expensive, and emits radiation (5). Some bone metabolism biochemical indicators, such as alkaline phosphatase (*ALP*), osteocalcin (*OCN*), N-terminal propeptide of type I collagen (*PINP*, a recommended biomarker of bone formation), β-isomerized C-terminal telopeptide of type I collagen (*β-CTX*), and parathyroid hormone (*PTH*), are also applied in the early diagnosis of osteoporosis, exhibiting high-sensitive, and strong-specific in reflecting bone homeostasis (6). However, owing to that these conventional biochemical indicators above are unstable inherently; the detection results of these biomarkers are not entirely reliable and stable. The mentioned problems of these existing osteoporosis diagnosis methods underscored the utmost importance for the identification of more specific and reliable biomarkers in the early diagnosis of osteoporosis.

Fortunately, over the past few decades, a novelty kind of RNA, microRNAs (miRNAs) have attracted tons of attention from researchers and clinicians. miRNAs, endogenous, single-stranded non-coding RNA (~20 nucleotides long), regulate gene expressions at post-transcriptional level through recognizing the complementary miRNA recognition element (MRE) or seed-matched sequences located in the 3’-untranslated region (UTR) of mRNAs (7). Despite that miRNAs share several sources of variability with enzyme/peptide markers, such as biological factors, technical issues, standardization of methods, and the use of internal controls, multiple researches have been conducted to measure the sources of miRNA variability and explore effective strategies to improve the detection power of the changes in miRNA expression (8, 9). Moreover, a great deal of researches showed that abnormal expression of miRNAs was closely associated with the occurrence of various diseases, such as cancers, Alzheimer’s disease, and bone metabolic diseases, especially in osteoporosis (10–12). During the past decades, there were already more than 200 articles directly working on miRNAs in osteoporosis. For example, Wang et al. found that miR-133a was a potential biomarker in circulating monocytes for postmenopausal osteoporosis (13). Another study revealed that miR-218 exerted a negative regulatory role in osteoclastogenesis and bone resorption by suppressing the p38MAPK-c-Fos-NFATc1 pathway (14). In addition, Bedene and his colleagues identified the positive regulatory role of miR-148a-3p in osteoclast differentiation and bone homeostasis (15). All these studies hinted at the potential indicative roles of miRNAs in the diagnosis of osteoporosis. However, the regulatory effects of miRNAs involved in the bone homeostasis of osteoporosis have not been systematically explored until now.

Herein, we attempted to carry out an integrated bioinformatics analysis to identify the expression profiles of miRNAs and their potential targets in osteoporosis, which may provide novel miRNA profiles as diagnostic biomarkers for osteoporosis.

## Materials and Methods

### Literature Retrieval Strategy

Wide-scale literature retrieval was conducted on PubMed, Springer Link, Web of Science and CNKI (China National Knowledge Infrastructure) from December 2008 to December 2019. Literature searching full-process consists of four steps: First, to be as inclusive as possible, single terms such as microRNAs, miRNAs, mirna*, or microrna* were applied to obtain all miRNAs-related studies from the databases mentioned above. The same retrieval approach was used for osteoporosis: terms including “osteoporosis” and other more specific terms such as “osteoblasts”, “osteoclasts”, “low bone mineral density (BMD)”, “bone loss”, or “bone quality” were used as the keywords. Second, a combination of controlled vocabulary and text words were applied to retrieve literatures concerned miRNAs in osteoporosis by entering “terms relevant to osteoporosis” AND “microRNAs-related text” in the search box of the databases. Despite the adopted systematic search strategy, some relevant articles still had not been included. Third, to ensure the comprehensive of this analysis, some missing additional articles were added into the list of included literatures and labeled as ‘not identified from search strategy’ for transparency. Finally, to facilitate the collection and consultation of information, full-texts of all retrieved results were downloaded into Endnote (EndNote X8, Bld 7072, Thomson Research Soft, Stamford), and duplicates were removed.

### Selection Criteria and Data Extraction

To further screen the eligible literatures, titles, abstracts, and, if necessary, full texts of the initially retrieved references were manually evaluated by two authors independently with specific inclusion and exclusion criteria as outlined below: (1) investigated the expression of miRNAs in bone tissue or serum of osteoporosis patients and/or control group participants, (2) osteoporosis in the studies was diagnosed through double energy X-ray absorption method (DXA) or radiographic, (3) the miRNAs were detected through microarray analysis or validated by RT-PCR, (4) without language restrictions, including papers in Mandarin, and (5) was not a review, case reports, conference presentations, editorials, or expert opinion. All the data of miRNAs were extracted from the text, tables and figures of the retrieved literatures by two independent investigators. miRNAs highly associated with osteoporosis were screened by two steps: (1) *P*-value, a statistical index, representing the ratio of the number of osteoporosis-miRNA–related articles/the number of miRNA-related articles (as displayed in Eq. 1), was calculated to assess the bias and further appraise the correlation of miRNAs and osteoporosis (*P* < 0.05) (16); (2) an online web server: miRNA Functional Similarity v2.0 (MISIM v2.0, http://www.lirmed.com/misim/) (17) was applied to measure the associations of miRNAs and osteoporosis based on the weighted frequency values (weighted frequency > 7). Notably, the weighted frequency values in the “Disease-miRNA Association” column of MISIM v2.0 database were the weighting factors used to measure the correlation between miRNAs and specific diseases. The overlap of the miRNAs predicted from the above two methods was finally included in our study.

(1) $$P=1-\Sigma_{i-0}^{k-1}\;f(i)=1-\Sigma_{i=0}^{k-1}\frac{{(}_{i}^{K}){(}_{n-i}^{N-K})}{{}_{n}^{N}}$$

where N represents the total number of papers in the literature databases (25.8 million articles, from December 2008 to December 2019), K is the number of articles associated with osteoporosis in databases (126, 170 papers), n shows the number of articles of each miRNA, and k represents the number of articles corresponding miRNA on osteoporosis. P-value indicates the correlations between each miRNAs and osteoporosis (significant when *P* < 0.05).

### Bioinformatics Analysis of Osteoporosis-Related miRNAs

**Figure 1:**
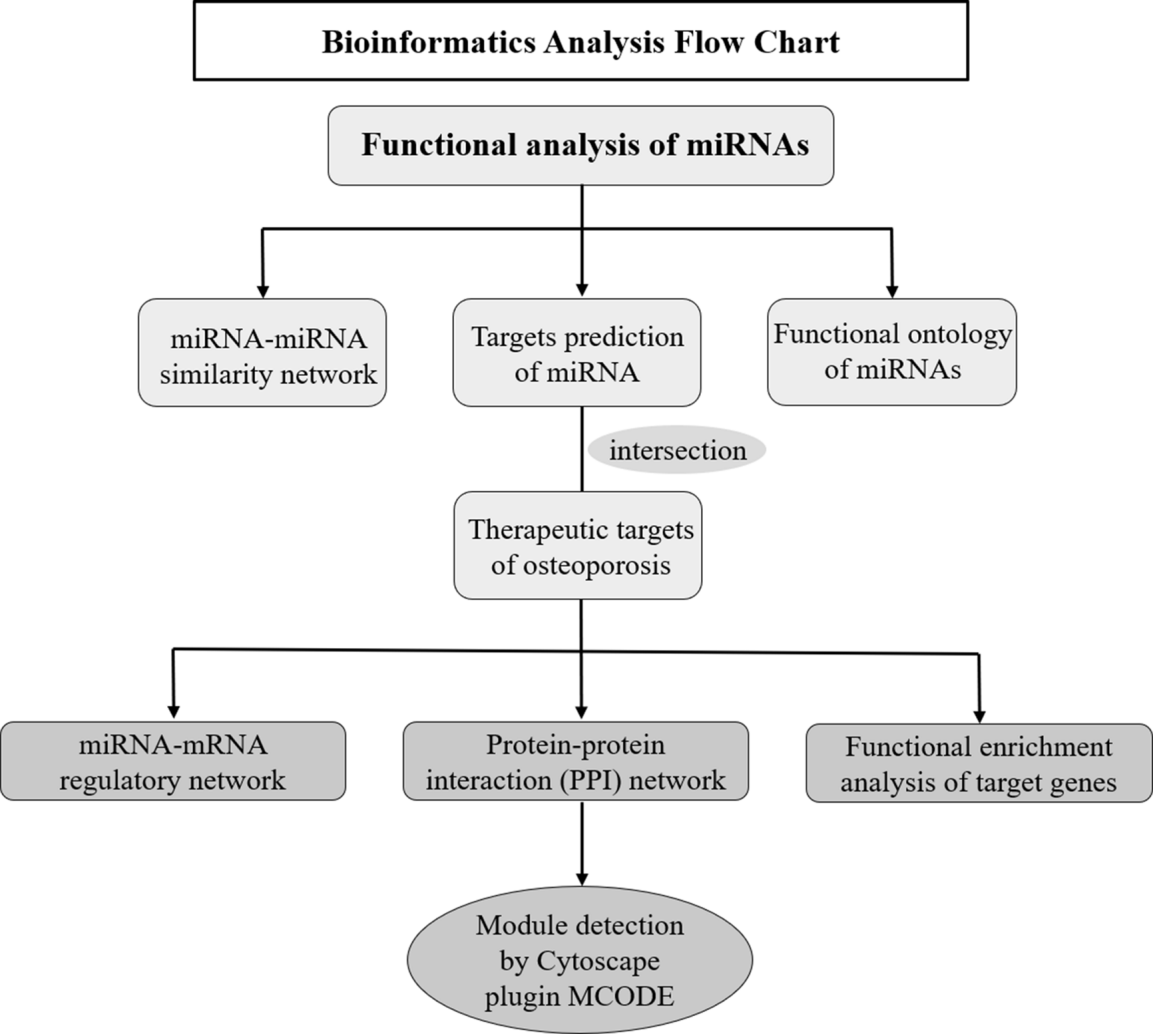
Flow diagram of the bioinformatics analysis.

Multiple bioinformatics methods were applied to explore the potential roles and expression profiles of miRNAs and their target genes in osteoporosis. Firstly, the functional similarity analysis and enrichment analysis of these miRNAs was conducted in online website MISIM v2.0. The miRNA-miRNA similarity network was generated by Cytoscape software, which was an open software project for biological network visualization (http://www.cytoscape.org/) (18). Secondly, target genes of these miRNAs were predicted employing miRWalk 3.0 database (19) (http://zmf.umm.uni-heidelberg.de/apps/zmf/mirwalk), which integrates 11 other databases: miRanda, miRDB, Targetscan, RNA22, miRBridge, etc. Meanwhile, the therapeutic targets of osteoporosis were obtained from six human disease therapeutic target databases, including GeneCards, Therapeutic target database (TTD), Disgenet database, Online mendelian inheritance in man (OMIM), Skeleton Genetics database, and Pharmacogenetics and pharmacogenomics knowledge base (PharmGKB). The cross part between the miRNA target genes and therapeutic targets of osteoporosis were identified as the osteoporosis-related genes of these miRNAs. Thirdly, miRNA-mRNA network was generated by Cytoscape software to investigate the regulatory relationship of miRNAs and their targets. Then, STRING (https://string-db.org/cgi/input.pl), an online database resource search tool for the retrieval of interacting proteins (20), was used to build a protein-protein interaction (PPI) network, and a confidence score > 0.4 was defined as significant. The PPI interaction of miRNAs from STRING was visualization with Cytoscape software. Based on the above data, Cytoscape software Molecular Complex Detection (MCODE) package was used to perform the module analysis of the PPI network and screen the hub genes. The parameters of network scoring and cluster finding were set as follows: degree cut-off = 2, node score cut-off = 0.2, k-core = 2, and max depth = 100. Finally, to further identify the biological functions of these predicted targets, gene ontology (GO) and KEGG Pathway enrichment analysis were implemented by Metascape database: a gene annotation and analysis resource (http://metascape.org) (21). The bioinformatics analysis process was illustrated in **Figure 1**.

### Experimental Verification

#### (1) Hind-Limb Unloading Mice Model (Simulated Microgravity *In Vivo*)

Two-month-old male Balb/c mice were purchased from the Laboratory Animal Center of the Fourth Military Medical University (Xi’an, Shaanxi, China). The mice were subjected to tail suspension with the hind limbs unloaded for four weeks as previously described (22). In brief, mice were kept in standard cages with maintained suspension position at about a 30° angle. This maneuver permitted the animals to have ad libitum access to food and water. The animal’s overall appearance, drinking and eating habits, and tail were checked two times per day. All the experimental procedures used in the present study were approved by the Animal Care and Experimental Safety of Northwestern Polytechnical University (23). (Note: The animal samples were kindly provided by the co-author Zhihao Chen.)

#### (2) RNA Extraction, Reverse Transcription, and RT-PCR

Quantitative reverse transcription-PCR was used to assess the mRNA expression levels of these miRNAs in osteoporosis mice model. Total RNA including miRNAs was isolated from the tibia samples without bone marrow of HLU mice and controls with E.Z.N.A.^®^ miRNA Isolation Kit (Omega, USA). The RNA concentration and purity were confirmed by the spectrophotometric ratio using absorbance measurements at wavelengths of 260 and 280 nm on a NanoDrop-2000 (Thermo Scientific, Waltham, Massachusetts, United States). Total RNA of 1μg was used for cDNA synthesis using Mir-X miRNA First-Strand Synthesis Kit according to the Mir-X miRNA First-Strand Synthesis User Manual (Takara Bio USA, Inc.). Quantitative PCR amplification was performed using the Thermal Cycler C-1000Touch system (BIO-RAD CFX Manager, Hercules, CA) and Mir-X miRNA TB Green *q*RT-PCR kit (Takara Bio USA, Inc.) according to the user manual. All amplifications were normalized to U6. Data were analyzed using the comparative Ct method (2^−ΔΔCt^) and expressed as fold change compared to corresponding control.

### Statistical Analysis

All experiments were independently repeated at least three times with tissues established in triplicate for each single assay. Statistical analyses of the data were performed using the GraphPad Prism 8 software (GraphPad Software, La Jolla, CA), and a student *t*-test was used. All data were reported as the mean ± standard deviation, and *P* < 0.05 was considered statistically significant.

## Results

### Differentially Expressed miRNAs in Osteoporosis

**Figure 2:**
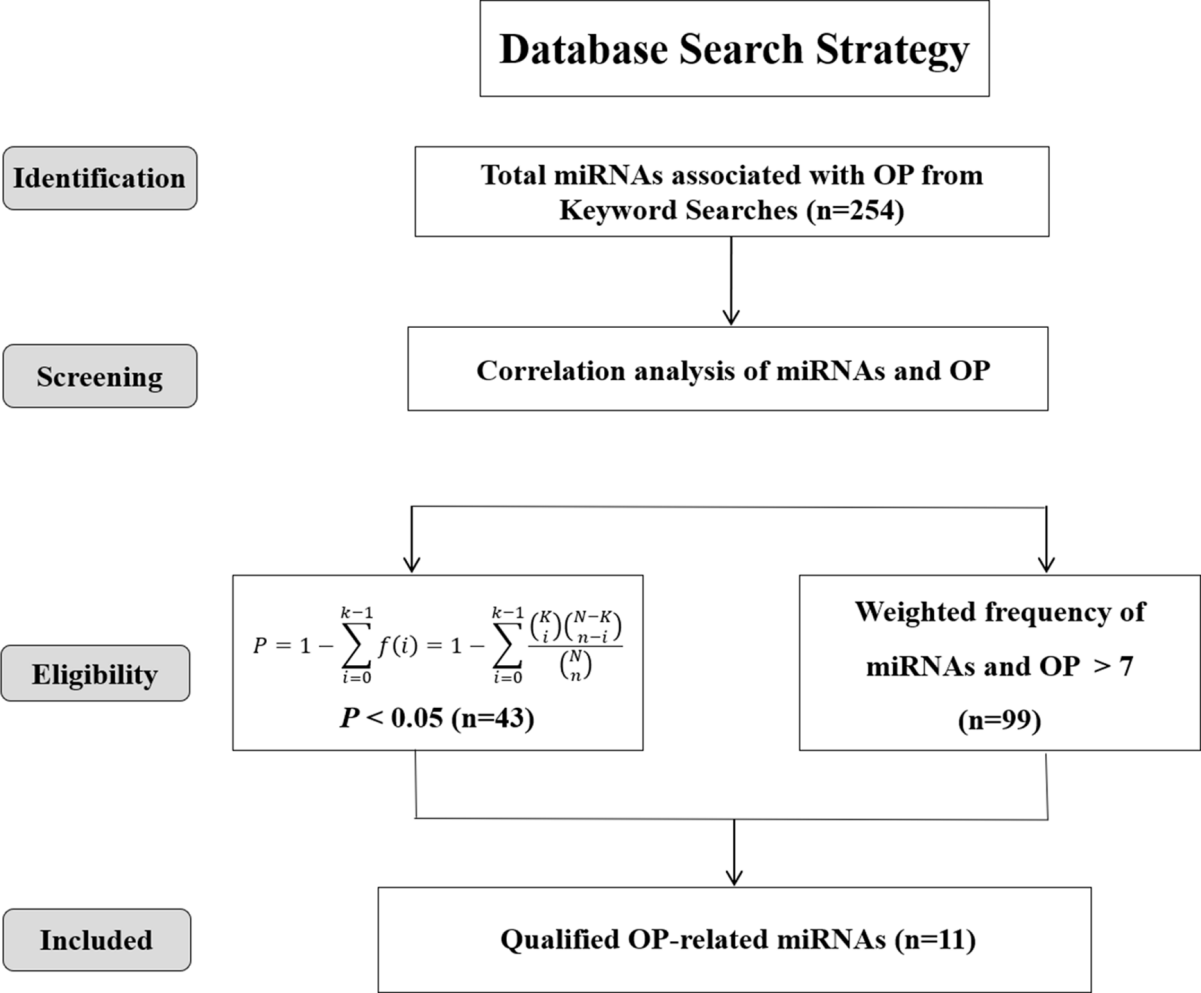
Screening strategy of osteoporosis-associated miRNAs.

A total of 254 miRNAs associated with osteoporosis were initially obtained through the data mining of the literature, which were retrieved from PubMed, Springer Link and Web of Science databases based on the used search terms. Then, based on the criterion of *P* < 0.05, 43 well-studied osteoporosis-related miRNAs were further screened out. Meanwhile, another 99 miRNAs were filtered out according to the weighted frequency indicating the correlation between miRNAs and osteoporosis in MISIM v2.0. Finally, a total of 11 eligible miRNAs were identified in the overlap of the results of above two screening steps and remained for further analysis. The screening process of these eligible miRNAs was shown in **Figure 2**.

**Figure 3:**
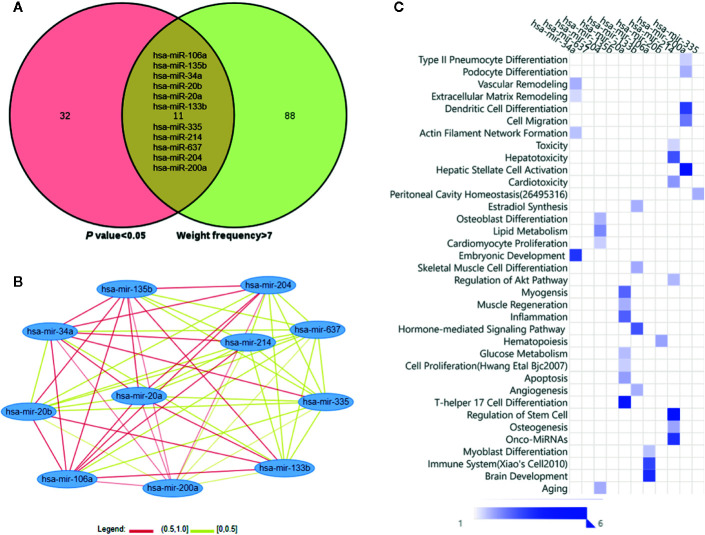
Osteoporosis-related miRNAs and their functional similarities and enrichment analysis. **(A)** Venn diagram of miRNAs based on the P value and weighted frequency. **(B)** The network of osteoporosis-related miRNAs based on functional similarities. **(C)** Functional enrichment analysis of osteoporosis-related miRNAs.

As shown in **Figure 3A**, the Venn diagram displayed the 11 most well studied miRNAs associated with osteoporosis (*P* < 0.05, weight frequency > 7). Detailed information of these 11 miRNAs was listed in **Table 1**. Therein, hsa-miR-335-5p possesses the optimal P value (*P* = 0.0005), followed by hsa-miR-204-3p (*P* = 0.00096) and hsa-miR-637 (*P* = 0.0011), indicating that they may be the top well studied miRNAs associated with osteoporosis. Analogously, for the weighted frequency of miRNAs, the top 3 miRNAs were hsa-miR-20a-5p (weighted frequency = 8.5901), hsa-miR-133b (weighted frequency = 8.5393), and hsa-miR-106a-5p (weighted frequency = 8.4640), which suggested that they might be the miRNAs most associated with osteoporosis (**Table 1**). Moreover, the detail information of the 11 miRNAs including the patient characteristics and detection assays were listed in the **Table 2**.

**Table 1:** Details of computationally predicted osteoporosis responsive miRNAs.

Identified miRNAs	Mimat ID	Mature miRNA Sequence	Weighted frequency	*P* value
hsa-miR-20a-5p	MIMAT0000075	UAAAGUGCUUAUAGUGCAGGUAG	8.5901	0.0044**
hsa-miR-133b	MIMAT0000770	UUUGGUCCCCUUCAACCAGCUA	8.5393	0.0041**
hsa-miR-106a-5p	MIMAT0000103	AAAAGUGCUUACAGUGCAGGUAG	8.4640	0.0412*
hsa-miR-135b-5p	MIMAT0000758	UAUGGCUUUUCAUUCCUAUGUGA	8.4420	0.0170*
hsa-miR-34a-5p	MIMAT0000255	UGGCAGUGUCUUAGCUGGUUGU	8.4147	0.0281*
hsa-miR-204-5p	MIMAT0000265	UUCCCUUUGUCAUCCUAUGCCU	8.0934	0.00096**
hsa-miR-637	MIMAT0003307	ACUGGGGGCUUUCGGGCUCUGCGU	8.0265	0.0011**
hsa-miR-214-3p	MIMAT0000271	ACAGCAGGCACAGACAGGCAGU	7.9913	0.0036**
hsa-miR-200a-3p	MIMAT0000682	UAACACUGUCUGGUAACGAUGU	7.8642	0.0382*
hsa-miR-20b-5p	MIMAT0001413	CAAAGUGCUCAUAGUGCAGGUAG	7.7692	0.0311*
hsa-miR-335-5p	MIMAT0000765	UCAAGAGCAAUAACGAAAAAUGU	7.2249	0.0005**

Weighted frequency refers to the correlation between these miRNAs and osteoporosis; P value refers to the ratio of the number of osteoporosis-miRNA–related articles/the number of miRNA-related articles, *P < 0.05, **P < 0.01.

**Table 2:** Details of the included osteoporosis-associated miRNAs.

Identified miRNAs	Tissue	Patients	Detection Method
hsa-miR-20a-5p	Bone tissue	Postmenopausal women with osteoporotic fractures	quantitative RT-PCR
hsa-miR-133b	Serum	Postmenopausal women with osteoporotic fractures	quantitative RT-PCR
hsa-miR-106a-5p	Serum	Postmenopausal women with osteoporotic fractures	quantitative RT-PCR
hsa-miR-135b-5p	Bone tissue	Osteoporosis patients	quantitative RT-PCR
hsa-miR-34a-5p	Serum	Osteoporosis patients caused by impaired WNT1	quantitative RT-PCR
hsa-miR-204-5p	Bone tissue	Females with osteoporotic fractures	microarray analysis
hsa-miR-637	Serum	Female osteoporotic patients	quantitative RT-PCR
hsa-miR-214-3p	Bone tissue	Aged patients with osteoporotic fracture	quantitative RT-PCR
hsa-miR-200a-3p	Serum	Osteoporosis patients	quantitative RT-PCR
hsa-miR-20b-5p	Serum	Females with X-linked PLS3-related osteoporosis	quantitative RT-PCR
hsa-miR-335-5p	Serum	Patients with idiopathic osteoporosis	quantitative RT-PCR

In **Figure 3B**, the miRNA-miRNA similarity network was generated with the miRNA pairs based on their similarity coefficients, showing the multiple functional interactions among these miRNAs. Specifically, 55 miRNA similarity (MISIM) pairs of these 11 miRNAs were obtained, 44% miRNA pairs possess MISIM higher than 0.5, i.e., the red edges in the network. Therein, hsa-mir-20a and hsa-mir-34a have the optimal functional similarity (Similarity = 0.62), followed by the miRNA pairs between hsa-mir-214 and hsa-mir-34a (Similarity = 0.60), and hsa-mir-20a and hsa-mir-135b (Similarity = 0.60). Furthermore, the functional enrichment analysis showed that these 11 miRNAs were primarily associated with significant pathways including dendritic cell differentiation, osteoblast differentiation, skeletal muscle cell differentiation, inflammation, Th17 cell differentiation, immune system, aging, and so on (**Figure 3C**).

### Target Genes of miRNAs and miRNA-mRNA Regulatory Network

**Figure 4:**
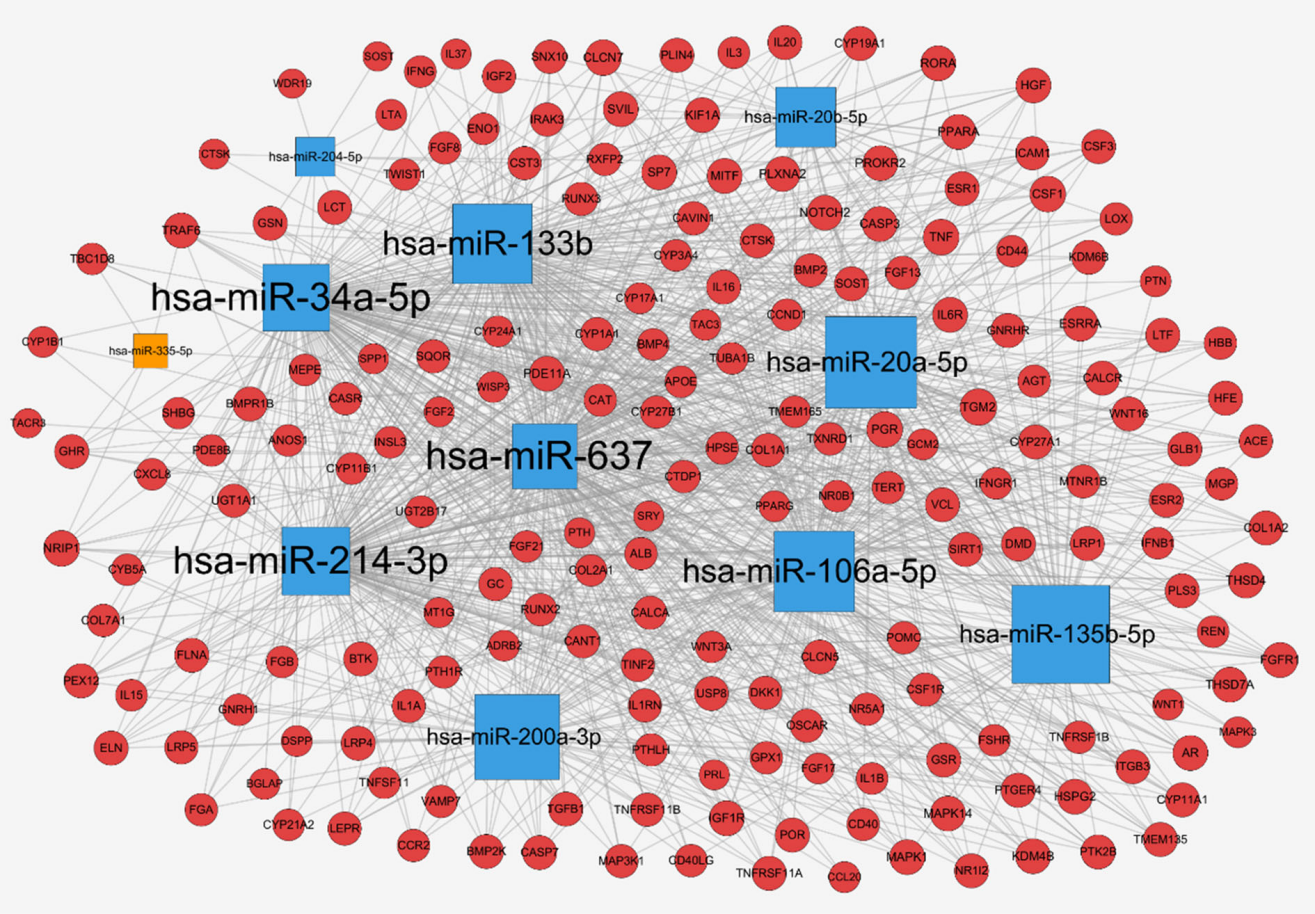
miRNA-mRNA network for miRNAs associated with osteoporosis: up-regulated miRNAs (blue square), down-regulated miRNAs (orange square), target genes (red circle), and the node size was proportional to its degree.

To further explore the regulation of miRNAs in osteoporosis progression, a total of 2752 target genes of these 11 differentially expressed miRNAs were predicted from miRWalk 3.0 online database. Meanwhile, 413 therapeutic targets of osteoporosis were obtained from six human disease therapeutic target databases, including GeneCards, therapeutic target database (TTD), Skeleton Genetics database, Online mendelian inheritance in man (OMIM), Disgenet database, and Pharmacogenetics and pharmacogenomics knowledge base (PharmGKB). By the integration of both the miRNA target genes and therapeutic targets of osteoporosis, totally, 198 genes were identified as osteoporosis-related target genes of these miRNAs. Then, the miRNA-mRNA regulatory network was generated with 209 nodes (11 miRNAs and their 198 osteoporosis-related target genes) and 1,079 edges (**Figure 4**), in which the node size was proportional to its degree. In the network, most of miRNAs were connected to multiple targets with an average degree of 18 each miRNA, among of which hsa-miR-637 possesses the highest number of targets (N = 171), followed by hsa-miR-34a with 166 targets and hsa-miR-133b (N = 133). Meanwhile, we noticed that many genes were common targets for different miRNAs. Statistically, about 55% of genes were targeted by more than 6 miRNAs. For example, Transglutaminase 2 (TGM2) were shared by 10 miRNAs except hsa-miR-335-5p; Progesterone Receptor (PGR), Colony Stimulating Factor 1 (CSF1) and Cathepsin K (CTSK) were targeted by 9 miRNAs; TNF Receptor Associated Factor 6 (TRAF6), Sclerostin (SOST), Androgen Receptor (AR) and Insulin Like Growth Factor 1 Receptor (IGF1R) were targeted by 8 miRNAs, revealing the multiple interactions of miRNAs and genes. Moreover, the 11 miRNAs have intricate associations with each other through targeting the common genes. Among of them, hsa-miR-637 and hsa-miR-34a shared 152 targets, with the greatest number of the common targets, followed by hsa-miR-34a and hsa-miR-214 with 143 common targets, and hsa-miR-34a and hsa-miR-20a with 92 common genes.

### Protein-Protein-Interaction (PPI) Network

**Figure 5:**
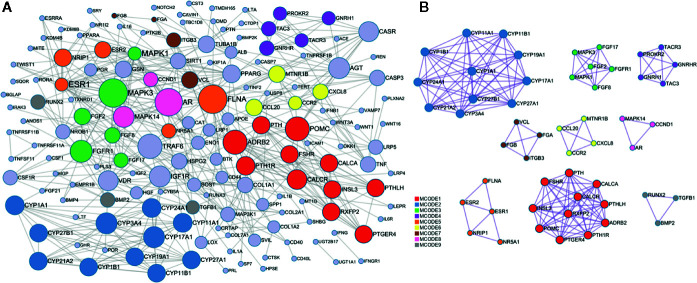
Protein-protein interaction (PPI) network of the target genes. **(A)** PPI network was constructed based on the target genes. Nodes in different colors represent the genes from different modules, the circle size indicates the connectivity of the genes. **(B)** The 9 most significant modules and networks.

The PPI network of the target genes of these miRNAs was generated based on the STRING online database, containing 160 nodes and 468 edges (**Figure 5A**), in which the node size was proportional to its degree. Among the 160 genes, the top 10 targets with high degree were identified as hub genes, they were adrenoceptor beta 2 (*ADRB2*), androgen receptor (*AR*), estrogen receptor 1 (*ESR1*), fibroblast growth factor receptor 1 (*FGFR1*), TNF receptor associated factor 6 (*TRAF6*), filamin A (*FLNA*), mitogen-activated protein kinase 1 (*MAPK1*), mitogen-activated protein kinase 3 (*MAPK3*), insulin like growth factor 1 receptor (*IGF1R*), and proopiomelanocortin (*POMC*). Specifically, *ESR1* possesses the most interactions with other genes (degree = 26), followed by *MAPK1* (degree = 24) and *FLAN* (degree = 20). To further investigate the associations between these genes, we found that these genes were mapped into nine modules by Cytoscape plugin MCODE with score > 5 (**Figure 5B**). In addition, functional enrichment analysis for these modules indicated that MCODE1 (module 1) was mainly associated with positive regulation of cytosolic calcium ion concentration, bone resorption and skeletal system development. MCODE2 (module 2) was typically involved in metabolic disorders of biological oxidation enzymes, ion binding and steroid hormone biosynthesis. MCODE3 (module 3) was primarily related to PI3K-Akt signaling pathway, fibroblast growth factor receptor signaling pathway and MAPK signaling pathway.

### Functional Enrichment Analysis of the Target Genes of miRNAs

**Figure 6:**
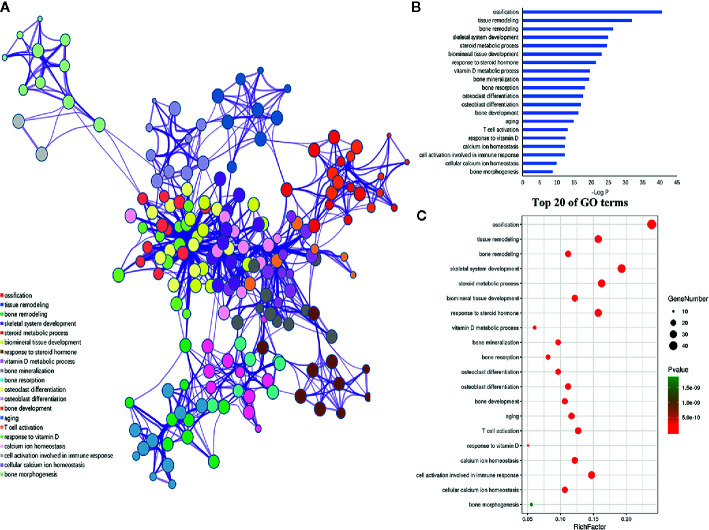
GO enrichment analysis of the putative genes of miRNAs. **(A)** Network of the enriched GO terms, which were colored by cluster ID, where nodes that share the same cluster ID were typically close to each other. **(B)** The top 20 enriched GO terms of target genes. **(C)** Bubble diagram of the enriched GO terms: the gradual color represents the P value; the size of the black spots represents the gene number.

**Figure 7:**
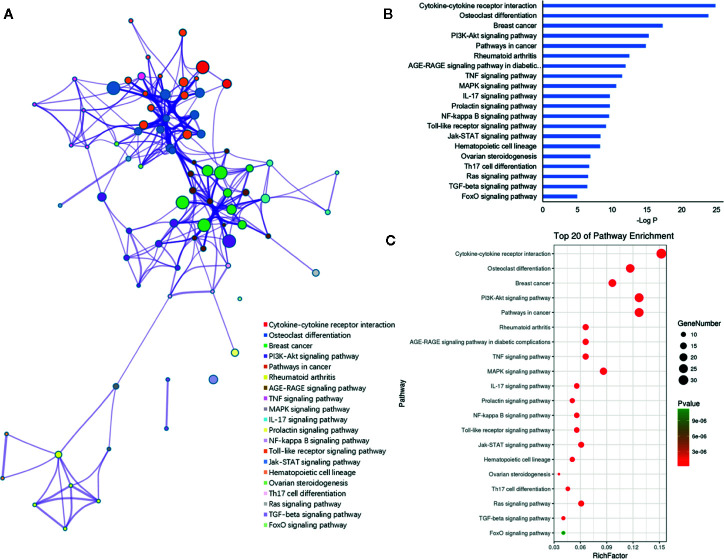
KEGG pathway enrichment analysis of the putative genes of miRNAs. **(A)** Network of enriched pathways colored by cluster ID, where nodes shared the same cluster ID was typically close to each other. **(B)** The top 20 enriched pathways of target genes. **(C)** Bubble diagram of the enriched pathways: the gradual color represents the P value; the size of the black spots represents the gene number.

Furthermore, the 198 target genes above were mapped into Metascape database for functional annotation analysis. The detailed information of GO terms and pathways of these targets were listed in **Tables S1**, **S2**. Here, biological processes with *P* < 0.05 were considered statistically significant. The terms network, bar graph and bubble diagram of the top 20 enriched GO terms were shown in **Figures 6A–C**, respectively, which displayed that the target genes were mainly enriched in biological processes associated with bone metabolism, such as ossification, bone remodeling, skeletal system development, BMP signaling pathway, bone mineralization, bone resorption, osteoclast differentiation and osteoblast differentiation. In addition, the pathway enrichment analysis revealed that these target genes were primarily mapped into cytokine-cytokine receptor interaction, osteoclast differentiation, PI3k-Akt signaling pathway, TNF signaling pathway, IL-17 signaling pathway, NF-κB signaling pathway, rheumatoid arthritis, and so on (**Figures 7A–C**). Strikingly, the top 20 enriched pathways were primarily related to immune regulation and bone metabolism. In the complex network of the first 20 clustering gene sets (**Figures 6A**, **7A**), the node size was in proportion to the gene number in the clustering term. These results suggested that the target genes were mainly involved in biological processes related to bone metabolism and immune regulation, which was in accordance with the functional enrichment analysis results of these 11 miRNAs (**Figure 3C**).

### Validation of the miRNA Expression in Osteoporosis

**Figure 8:**
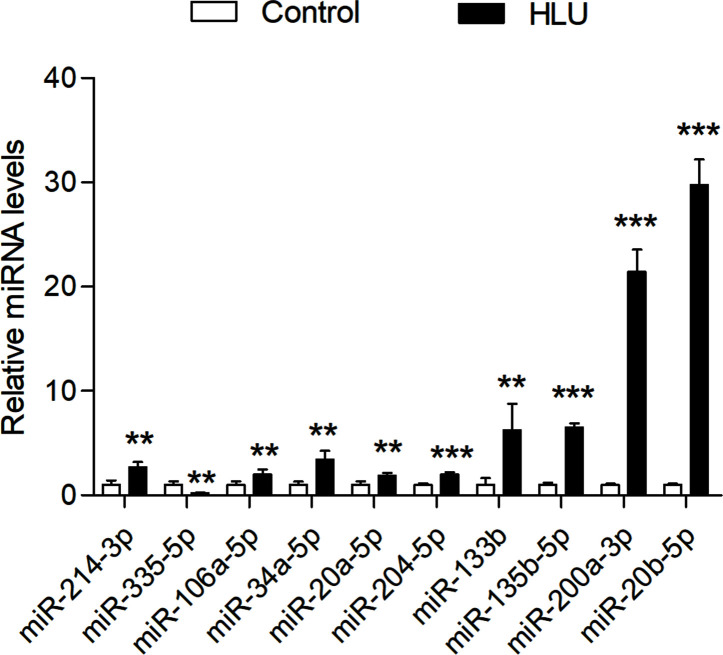
Validation of the expression profile of these miRNAs by RT-PCR between the HLU group (n = 6) and the control group (n = 6). All samples were normalized to the expression of hsa-miR-U6, and the relative expression levels of each miRNA were analyzed using the 2^−ΔΔCt^ method. **P < 0.01; ***P < 0.001.

The expression levels of these miRNAs were detected in tibia samples of HLU osteoporosis mice model and normal controls by RT-PCR (**Table 3** and **Figure 8**). Therein, the expression levels of miR-214-3p, miR-106a-5p, miR-133b, miR-135b-5p, miR-20a-5p, miR-20b-5p, miR-200a-3p, miR-34a-5p, and miR-204-5p were significantly increased in tibia samples of HLU mice, while miR-335-5p was down-regulated in HLU mice tibia samples compared with the controls. Due to that miR-637 is a primate-specific miRNA and not present in mice, its expression was not validated. All the screened miRNAs were validated to be differentially expressed in osteoporosis by experiment, indicating that they may be diagnosis biomarkers for osteoporosis, which was consistent with our analytical results.

**Table 3:** Expression profile of these miRNAs in tibia samples of hind limb unloading (HLU) mice.

Experimentally verified miRNAs	Expression	Log_2_ (Fold change)	*P* value
mmu-miR-20a-5p	up	1.00	0.0053**
mmu-miR-106a-5p	up	1.00	0.0034**
mmu-miR-204-5p	up	1.02	0.0002***
mmu-miR-214-3p	up	1.42	0.0018**
mmu-miR-34a-5p	up	1.78	0.0019**
mmu-miR-133b	up	2.65	0.0045**
mmu-miR-135b-5p	up	2.70	5.66E-06***
mmu-miR-200a-3p	up	4.42	2.44E-06***
mmu-miR-20b-5p	up	4.90	0.0007***
mmu-miR-335-5p	down	-2.24	0.0098**

**P < 0.01 or ***P < 0.001 and 1 < Log2 (Fold change) <−1 were statistically significant.

## Discussion

Osteoporosis is a common subclinical malady of the skeleton disease, which has been aroused considerable attention all over the world due to the growing number of osteoporosis patients (24). MiRNAs, a class of evolutionarily conserved endogenous non-coding RNAs, play important roles in diverse physiological and pathological processes, including cell differentiation, proliferation, apoptosis and cancer development (10). Given the crucial roles that miRNAs appear to exert in bone metabolism and bone homeostasis, exploring their potential has recently garnered increased interest (25, 26). Plenty of differentially expressed miRNAs have been identified in bone samples or serums of osteoporosis patients, which might provide clinical signals to the understanding of the complex mechanisms and the accurate diagnosis of osteoporosis. However, the systematic analysis of the regulatory roles of miRNAs in osteoporosis still remains poor. The present work performed a comprehensive bioinformatics analysis to identify the expression profiles of miRNAs and their target genes involved in osteoporosis, aiming to provide novel biomarkers for clinical diagnosis of osteoporosis.

By the literature mining and database retrieval, 11 miRNAs and their 198 osteoporosis-related genes were obtained. The miRNA-mRNA network of the miRNAs and their targets revealed that the 11 miRNAs associated with osteoporosis exhibit multiple interactions with multiple targets. In addition, we observed that genes in this network were also targeted by a set of miRNAs in common. For example, Li et al. observed that miR-34a inhibited osteoblast differentiation of human mesenchymal stem cell (HMSC) through regulating its plurality of targets including Jagged1 (*JAG1*), cyclin D1 (*CCND1*), cyclin-dependent kinase 4 and 6 (*CDK4* and *CDK6*), and E2F transcription factor 3 (*E2F3*) (27). Another study showed that miRNA-20a promoted osteogenic differentiation of HMSC by co-regulating its multiple targets peroxisome proliferator activated receptor γ (*PPARγ*), *BMP* and activin membrane bound inhibitor (*BAMBI*), and cysteine rich transmembrane BMP regulator 1 (*CRIM1*) (28), which was consistent with our study results very well. Additionally, majority of the target genes were shared by more than one miRNA, and over 55% of which were targeted by more than 6 miRNAs. For instance, dickkopf-1 (*DKK1*), a powerful antagonist of canonical WNT signaling pathway and biomarker for osteoporosis, was predicted as the shared targets of hsa-miR-133b, -20a-5p, -637, -214-3p, -106a-5p, and -135b-5p in our study. Coincidentally, some studies also revealed that DKK1 was targeted by miR-335-5p, miR-433-3p and miR-135b-5p commonly (29–31). Besides, another crucial osteoblast differentiation biomarker RUNX2 was regulated by a panel of miRNAs, including miR-204, -23a, -30c, -34a, -133a, and so on (32, 33). The PPI network resulted in an interesting finding that, *TRAF6*, *ADRB2*, *AR*, *ESR1*, *FGFR1*, *FLNA*, *MAPK1*, *MAPK3*, *IGF1R*, and *POMC* were identified as the top 10 hub genes owing to their higher connectivity, suggesting these genes may play important regulatory roles in the pathogenesis of osteoporosis. Take *TRAF6*, for example, which represents a molecular bridge spanning immune and skeleton (34), has been well studies for its crucial biological activities in osteoclast differentiation and immune response (35). Also, TRAF6 is the critical adaptor protein that transduces RANKL/RANK signaling, which accelerates osteoclast formation by binding with its two best-characterized interacting receptors CD40 and RANK, both of which play important roles in the regulation of immunity and bone resorption (36). Taken together, it appears that miRNAs and their targets are mutually coordinated to exert their broad biological roles in the regulation of osteoporosis.

Furthermore, the functional enrichment analysis showed that these miRNAs and their targets were primarily involved in biological processes associated with bone metabolism and immune regulation, such as bone remodeling, osteoclast differentiation, Toll like receptor signaling pathway, Th17 cell differentiation and IL-17 signaling pathway. As for the immune regulation, this result supports the prior observations that immune cells and immune cell-derived cytokines broadly participant in the development of osteoporosis, therefore, the immune response is another recognized mechanism of osteoporosis (37, 38).With respect to IL-17 signaling pathway, it serves as a bridge between immune system and skeletal system (39). IL-17 is a pleiotropic cytokine secreted by Th17 cells, which potently potentiates RANKL-induced osteoclastogenesis, resulting in intensifying bone loss (40). Some representative proinflammatory cytokines secreted by T cells, including *TNF-α*, interleukin-6 (*IL-6*), interleukin-17 (*IL-17*), CD40 ligand (*CD40L*), and receptor activator for NF-κB ligand (*RANKL*), were contributed to the bone loss under inflammation conditions through promoting the osteoclastogenesis (41–43). Notably, all these cytokines were the predicted targets of these miRNAs associated with osteoporosis in our study. For instance, the T cell costimulatory factor CD40L has been reported to intensify the dysregulation of bone resorption and formation under both estrogen deficiency and continuous PTH treatment through stimulating the additional production of RANKL and inhibiting the secretion of OPG, thus leading to osteoporosis (43, 44). In addition, Azizieh et al. found that TNF-α contributes to promoting bone resorption and repressing bone formation, which results in aggravated bone loss (45). Therefore, these results not only revealed the important regulatory roles of these miRNAs in osteoporosis but also conforms to the opinion that osteoporosis is a chronic disorder associated with immuno-inflammatory responses.

Given that the abnormal expression of most of these miRNAs have been identified in osteoporosis types such as postmenopausal osteoporosis, senile osteoporosis and glucocorticoid-induced osteoporosis (23, 46–49), here we selected the model of disuse osteoporosis to validated the expression of these miRNAs. Interestingly, various research groups who identified the differentially expressed miRNAs in osteoporosis have not come up with the exactly identical results. For example, Seeliger et al. identified that five miRNAs (miR-21, miR-23a, miR-24, miR-25, miR-100, and miR-125b) displayed an up-regulation both in serum and bone tissue of osteoporotic fracture patients (50). In distinction with the research of Seeliger, Yavropoulou and his colleagues found that the expressions of miR-21 and miR-23a were significantly lower in the serum of patients with osteoporotic fracture (51). Thus, these clues hinted that there was still a distinct lack of various osteoporosis animal models and clinical samples to thoroughly and deeply explore the different roles and internal mechanisms of these miRNAs in different types osteoporosis.

In summary, our study comprehensively explored the regulatory roles of miRNAs in osteoporosis based on the bioinformatics analysis, which may provide clues and possibilities for miRNAs as diagnostic biomarkers of osteoporosis as well as an innovative approach for the identification of biomarkers in other chronic diseases. Given the inconsistent findings of the existing researches, further studies were strongly necessary to thoroughly excavate the roles and potentials of these miRNAs in different types of osteoporosis, which may offer reliable clinical guidance for miRNAs as biomarker in osteoporosis diagnosis.

## Conclusion

In the present work, we comprehensively explored the regulatory roles of miRNAs in osteoporosis. Currently, 11 most associated with osteoporosis miRNAs were identified to be significantly differentially expressed in HLU mice osteoporosis model through data processing and RT-PCR validation, which may be potential as drug targets and diagnostic markers for osteoporosis. *TRAF6*, *ADRB2*, *AR*, *ESR1*, *FGFR1*, *FLNA*, *MAPK1*, *MAPK3*, *IGF1R*, and *POMC* were determined as the hub genes in the PPT network. Moreover, functional enrichment analysis demonstrated that these miRNAs and their target genes were mainly enriched in GO terms and pathways related to bone and immune system: GO terms like bone remodeling, skeletal system development, bone mineralization, T cell activation, and Th17 cell differentiation; pathways such as cytokine-cytokine receptor interaction, osteoclast differentiation, TNF signaling pathway, PI3K-Akt signaling pathway, rheumatoid arthritis, and IL-17 signaling pathway. These miRNAs were validated to be differentially expressed in the tibia samples of HLU mice by experiment. Given these results above, we foresee that miRNAs may be emerging as novel diagnostic biomarkers for osteoporosis.

## Data Availability Statement

The datasets analyzed in this article are not publicly available. Requests to access the datasets should be directed to huai_@mail.nwpu.edu.cn.

## Ethics Statement

The animal study was reviewed and approved by Lab Animal Ethics & Welfare Committee of Northwestern Polytechnical University.

## Author Contributions

Data curation: YH. Formal analysis: YH, WZ, and AQ. Experiment validation: ZC, FZ, and YH. Funding acquisition: AQ and WZ. Methodology: YH, WW, QH, and ML. Writing—original draft: YH. Writing—review and editing: WZ, KD, SJ, ZM, and CC. All authors contributed to the article and approved the submitted version.

## Funding

This work was supported by the National Natural Science Foundation of China under No. 81901917 and No. 31570940; the Fundamental Research Funds for the Central Universities under No. 3102017OQD050; China’s Post-doctoral Science Fund under No. 2017M623249; the Key Research and Development Project of Shaanxi Province under No. 2018SF-363; and the grant under BKJ17J004.

## Conflict of Interest

The authors declare that the research was conducted in the absence of any commercial or financial relationships that could be construed as a potential conflict of interest.
