# Supplementary material for: A Comprehensive Analysis of MicroRNAs in Human Osteoporosis
Source: Front Endocrinol (Lausanne). 2020 Oct 21;11:516213. doi: 10.3389/fendo.2020.516213 (PMC7609919; doi:10.3389/fendo.2020.516213)
Supplement: Supplementary file 1 [file DataSheet_1.pdf]

Table S1. Information of GO terms of target genes.

Term	Description	Gene count	*P* value
GO:0001503	ossification	47	2.65E-41
GO:0048771	tissue remodeling	31	1.53E-32
GO:0046849	bone remodeling	22	4.03E-27
GO:0001501	skeletal system development	38	1.29E-25
GO:0008202	steroid metabolic process	32	2.52E-25
GO:0031214	biomineral tissue development	24	1.01E-23
GO:0048545	response to steroid hormone	31	4.29E-22
GO:0042359	vitamin D metabolic process	12	2.89E-20
GO:0030282	bone mineralization	19	4.19E-20
GO:0045453	bone resorption	16	7.72E-19
GO:0030316	osteoclast differentiation	19	2.33E-18
GO:0001649	osteoblast differentiation	22	1.09E-17
GO:0060348	bone development	21	5.83E-17
GO:0007568	aging	23	1.31E-15
GO:0042110	T cell activation	25	7.69E-14
GO:0033280	response to vitamin D	10	3.26E-13
GO:0055074	calcium ion homeostasis	24	5.77E-13
GO:0002263	cell activation involved in immune response	29	5.83E-13
GO:0006874	cellular calcium ion homeostasis	21	1.25E-10
GO:0060349	bone morphogenesis	11	1.82E-09
GO:0030509	BMP signaling pathway	10	4.76E-07
GO:0016055	Wnt signaling pathway	18	1.99E-10

$P < 0.01$ was considered as significant enriched.

Table S2. Information of signaling pathways of target genes.

Term	KEGG pathways	Gene count	*P* value
hsa04060	Cytokine-cytokine receptor interaction	30	1.43E-25
hsa04380	Osteoclast differentiation	23	1.46E-24
hsa05224	Breast cancer	19	5.81E-18
hsa04151	PI3K-Akt signaling pathway	25	5.58E-16
hsa05200	Pathways in cancer	25	1.46E-15
hsa05323	Rheumatoid arthritis	13	3.45E-13
hsa04933	AGE-RAGE signaling pathway	13	1.22E-12
hsa04668	TNF signaling pathway	13	3.82E-12
hsa04010	MAPK signaling pathway	17	2.74E-11
hsa04657	IL-17 signaling pathway	11	2.19E-10
hsa04917	Prolactin signaling pathway	10	2.19E-10
hsa04064	NF-kappa B signaling pathway	11	2.77E-10
hsa04620	Toll-like receptor signaling pathway	11	7.43E-10
hsa04630	Jak-STAT signaling pathway	12	4.95E-09
hsa04640	Hematopoietic cell lineage	10	5.78E-09
hsa04913	Ovarian steroidogenesis	7	1.39E-07
hsa04659	Th17 cell differentiation	9	2.01E-07
hsa04014	Ras signaling pathway	12	3.13E-07
hsa04350	TGF-β signaling pathway	8	3.72E-07
hsa04068	FoxO signaling pathway	8	1.14E-05

$P < 0.01$ was considered as significant enriched.

Table S3. Forward Primer Sequences of miRNAs for RT-PCR.

miRNAs	Primer Sequence (5'-3')
mmu-miR-34a-5p	caTGGCAGTGTCTTAGCTGGTTGT
mmu-miR-204-5p	ACGCTTCCCTTTGTCATCCTATG
mmu-miR-214-3p	TCTACAGCAGGCACAGACAGG
mmu-miR-20a-5p	ACGCTAAAGTGCTTATAGTGCAG
mmu-miR-106a-5p	gcCAAAGTGCTAACAGTGCAGGT
mmu-miR-133b	cTTTGGTCCCCTTCAACCAGCTA
mmu-miR-135b-5p	GCTATGGCTTTTCATTCCTATGTG
mmu-miR-200a-3p	GCTAACACTGTCTGGTAACGATG
mmu-miR-20b-5p	AGTGCAAAGTGCTCATAGTGCAG
mmu-miR-335-5p	GCGCCTCAAGAGCAATAACGAAA
